# Co(II) and Cd(II) Complexes Derived from Heterocyclic Schiff-Bases: Synthesis, Structural Characterisation, and Biological Activity

**DOI:** 10.1155/2013/754868

**Published:** 2013-08-21

**Authors:** Riyadh M. Ahmed, Enaam I. Yousif, Mohamad J. Al-Jeboori

**Affiliations:** Department of Chemistry, College of Education, (Ibn Al-Haitham), University of Baghdad, P.O. Box 4150, Adhamiya, Baghdad, Iraq

## Abstract

New monomeric cobalt and cadmium complexes with Schiff-bases, namely, *N*′-[(*E*)-(3-hydroxy-4-methoxyphenyl)methylidene]furan-2-carbohydrazide (L^1^) and *N*′-[(*E*)-(3-hydroxy-4-methoxyphenyl)methylidene]thiophene-2-carbohydrazide (L^2^) are reported. Schiff-base ligands L^1^ and L^2^ were derived from condensation of 3-hydroxy-4-methoxybenzaldehyde (iso-vanillin) with furan-2-carboxylic acid hydrazide and thiophene-2-carboxylic acid hydrazide, respectively. Complexes of the general formula [M(L)_2_]Cl_2_ (where M = Co(II) or Cd(II), L = L^1^ or L^2^) have been obtained from the reaction of the corresponding metal chloride with the ligands. The ligands and their metal complexes were characterised by spectroscopic methods (FTIR, UV-Vis, ^1^H, and ^13^C NMR spectra), elemental analysis, metal content, magnetic measurement, and conductance. These studies revealed the formation of four-coordinate complexes in which the geometry about metal ion is tetrahedral. Biological activity of the ligands and their metal complexes against gram positive bacterial strain *Bacillus* (G+) and gram negative bacteria *Pseudomonas* (G−) revealed that the metal complexes become less resistive to the microbial activities as compared to the free ligands.

## 1. Introduction

Schiff-base ligand is an interesting class of compounds which have played a key role in the development of coordination chemistry. Schiff-bases and their complexes have a variety of applications in the biological systems and industry [1–6]. Furthermore, Schiff-bases are very important materials for inorganic chemists as these are widely used in medicinal inorganic chemistry due to their diverse biological, pharmacological, antitumor activities and their excellent chelating ability. Schiff-bases have gained much importance in catalysis, biomimetic modelling applications, designing molecular magnet molecules, and in liquid crystals aspect [7–9]. Schiff-base ligands with heterocyclic molecule and/or containing heteroatoms such as N, O, and S show a broad biological activity and are of special interest because of the variety of ways in which they are interacted to transition metal ions [10, 11]. Experimental studies related to DNA binding and cleavage were explored using a range of potent Cu(II) Schiff-base complexes. In addition, Schiff-base nickel(II) complexes have been regarded as models for enzymes such as urease [12]. Vanilline and furfurylamine Schiff-base derivatives are very useful biochemical materials having biological activities [13, 14]. Structural characterisation of Schiff-bases and their metal complexes are well documented, including X-ray molecular structure [15–19]. In this paper, we report the synthesis of new Schiff-bases L^1^ and L^2^, namely, *N*′-[(*E*)-(3-hydroxy-4-methoxyphenyl)methylidene]furan-2-carbohydrazide and *N*′-[(*E*)-(3-hydroxy-4-methoxyphenyl)methylidene]thiophene-2-carbohydrazide, respectively, and their metal complexes with Co(II) and Cd(II) ions.

## 2. Experimental

### 2.1. Materials

All reagents were commercially available and used without further purification. Solvents were distilled from appropriate drying agents immediately prior to use.

### 2.2. Physical Measurements

Melting points were obtained on a Buchi SMP-20 capillary melting point apparatus and are uncorrected. FTIR spectra were recorded as KBr disc using a Shimadzu 8400 FTIR spectrophotometer in the range 4000–400 cm^−1^. Electronic spectra of the prepared compounds were measured in the region 250–1100 nm for 10^−3^ M solutions in DMSO at 25°C using a Shimadzu 160 spectrophotometer with 1.000 ± 0.001 cm^−1^ matched quartz cell. ^1^H and ^13^C NMR spectra were acquired in DMSO-d_6_ solution using a Brucker AMX 400 MHz spectrometer with tetramethylsilane (TMS) as an internal standard for ^1^H NMR. Metals were determined using a Shimadzu (A.A) 680 G atomic absorption spectrophotometer. Chloride was determined using potentiometer titration method on a 686-Titroprocessor, 665 Dosimat Metrohm, Swiss. Conductivity measurements were made with DMSO solutions using a PW 9526 digital conductivity meter and room temperature magnetic moments were measured with a magnetic susceptibility balance (Johnson Matthey Catalytic System Division).

## 3. Synthesis

### 3.1. Preparation of *N′-[(E)*-(3-Hydroxy-4-methoxyphenyl)methylidene]furan-2-carbohydrazide (L^1^)

A solution of 3-hydroxy-4-methoxy-benzaldehyde (1.00 g, 6.572 mmoL) in methanol (5 mL) was added to a mixture of furan-2-carboxylic acid hydrazide (0.644 g, 6.572 mmoL) in methanol (5 mL), and then (2–4) drops of glacial acetic acid was added. The reaction mixture was refluxed for 4 h, filtered off, and then cooled to RT. Solvent was allowed to slow evaporation and a pale yellow solid was obtained. Yield (0.93 g, 54%), m.p = 183°C. IR data (cm^−1^): 3473 *ν* (O–H), 3202 *ν* (N–H), 1664 *ν* (C=O), 1629 *ν* (C=N). NMR data (ppm), ^1^H NMR data showed peaks at *δ*_H_ (400 MHz, DMSO-d_6_): 3.84 (3H, s, CH_3_), 6.92 (H, NH), 7.214–7.340 (3H, Ar-H), 7.85–7.911 (3H, Ar-H), 8.57 (1H, s, O–H), 8.74 (1H, s, H-C=N). ^13^C NMR data showed peaks at *δ*_*c*_ (100.63 MHz, DMSO-d_6_): 56.51 (O–CH_3_), (146.09; 109.41; 111.16 and 147.24) assigned to carbon of furan ring, (122.68; 115.05; 126.53; 148.51; 112.48 and 115.92) assigned to aromatic ring carbon, 149.50 (HC=N), 154.51 (C=O).

### 3.2. Preparation of *N′-[(E)*-(3-Hydroxy-4-methoxyphenyl)methylidene]thiophene-2-carbohydrazide (L^2^)

The method used to prepare L^2^ was similar to that used for L^1^ but thiophene-2-carboxylic acid hydrazide (1.00 g, 0.007 mmoL) was used in place of furan-2-carboxylic acid hydrazide. The quantities of other reagents used were adjusted accordingly. An identical work-up procedure gave the title compound as an orange-yellow solid. Yield (1.27 g, 65%), m.p. = 170°C. IR data (cm^−1^): 3375 *ν* (O–H), 3163 *ν* (N–H), 1669 *ν* (C=O), 1618 *ν* (C=N). NMR data (ppm), *δ*_H_ (400 MHz, DMSO-d_6_): 3.83 (3H, s, CH_3_), 6.69 (H, NH), 6.83–6.95 (3H, Ar-H), 7.06–7.40 (3H, Ar-H), 7.92 (H, s, O–H), 8.34 (H–C=N–). ^13^C NMR data *δ*_*c*_ (100.63 MHz, DMSO-d_6_): 56.30 (O–CH_3_), (129.59; 128.65; 129.59; and 137.70) assigned to carbon of furan ring, (116.11; 124.50; 125.37; 123.09; 132.18 and 148.58) assigned to aromatic ring carbon, 161.34 (C=O), 151.86 (H−C=N).

### 3.3. General Synthesis of Complexes

A methanolic solution (10 mL) of the hydrated metal salt (1 mmoL), MCl_2_·XH_2_O (where M = Co^II^; X = 6; Cd^II^; X = 2), was stirred into methanolic solution of the Schiff-base ligand (2 mmoL) in methanol (15 mL). The reaction mixture was then refluxed for 2 h to give a coloured precipitate which was collected by filtration, washed with cold ethanol (5 mL), and dried at room temperature. Elemental analysis data, colours, and yields for the complexes are given in Table 1. 

### 3.4. Determination of Bacteriological Activity

Bioactivities were investigated using agar-well diffusion method [20]. The wells were dug in the media with the help of a sterile metallic borer with centers at least 24 mm. Recommended concentration (100 **μ**L) of the test sample 1 mg/mL in DMSO was introduced in the respective wells. The plates were incubated immediately at 37°C for 20 h. Activity was determined by measuring the diameter of zones showing complete inhibition (mm). In order to clarify the role of DMSO in the biological screening, separate studies were carried out with the solutions alone of DMSO and they showed no activity against any bacterial strains. Ligand found to be potentially active against these bacterial strains were compared with its complexes.

## 4. Results and Discussion

### 4.1. Chemistry

**Scheme 1:**
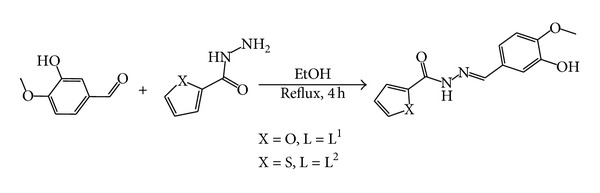
Synthesis diagram of the Schiff-base ligands (L^1^ and L^2^).

**Scheme 2:**
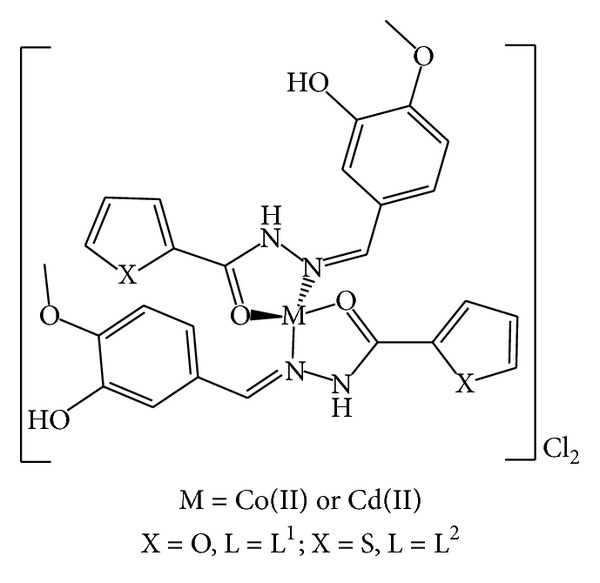
Proposed structure of complexes.

The reaction of 3-hydroxy-4-methoxy-benzaldehyde with furan-2-carboxylic acid hydrazide or thiophene-2-carboxylic acid hydrazide in mole ratio 1 : 1 gave L^1^ and L^2^, respectively (Scheme 1). The Schiff-bases were characterised by elemental analysis (Table 1), IR (Table 2), UV–Vis spectroscopy (Table 3) and ^1^H-, and ^13^C-NMR spectra. The dielectrolyte metal-complexes were synthesised by mixing at reflux 2 mmole of the Schiff-base ligand with 1 mmole of the appropriate metal chloride. Monomeric complexes of the general formulae [M(L)_2_]^+2^ (where M = Co^II^ and Cd^II^) were obtained (Scheme 2). The complexes are air-stable solids, soluble in DMSO and DMF. The complexes are sparingly soluble in MeOH and not soluble in other common organic solvents. The coordination geometries of the complexes were deduced from their spectra. The analytical data (Table 1) agree well with the suggested formulae. Conductivity measurements of the complexes in DMSO solutions lie in the 71.4–78.1 cm^2^ Ω^−1^ mol^−1^ range, indicating their 1 : 2 electrolytic behaviour (Table 1) [21]. 

### 4.2. FTIR and NMR Spectra

The important infrared bands for the ligands and their complexes together with their assignments are listed in (Table 2). The IR spectra of the ligands show characteristic bands at 1664–1669 and 1629–1618 cm^−1^ due to the *ν* (C=O), *ν* (C=N) functional groups, respectively. The bands at 3473, 3203, and 3375, 3163 cm^−1^ assigned to the *ν* (O–H) and *ν* (N–H) stretching group for ligands L^1^ and L^2^, respectively, [22–24]. The IR spectra of the complexes exhibited ligand bands with the appropriate shifts due to complex formation (Table 2). The *ν* (C=O) and *ν* (C=N) stretching bands that appeared in the free ligands at ca. 1660 and 1620 cm^−1^, respectively, are shifted to lower frequency in the complexes and observed in the ranges 1641–1649 cm^−1^ and 1600–1613 cm^−1^ for *ν* (C=O) and *ν* (C=N), respectively. These bands are assigned to a *ν* (C=O) and *ν* (C=N) stretches of reduced bond order. This can be attributed to delocalisation of metal electron density (*t*_2*g*_) to the *π*-system of the ligand [25, 26], indicating coordination of oxygen of C=O and nitrogen of the C=N moieties to the metal atoms [27]. The bands of *ν* (C–O) at ca. 1270 cm^−1^ in the free ligands are shifted to lower frequencies and appeared at 1203–1263 cm^−1^ for the complexes. At lower frequency the complexes exhibited bands around 540–549 and 404–416 cm^−1^ which could be assigned to *ν* (M–O) and *ν* (M–N) vibration mode [25]. These bands indicated that the imine, nitrogens and the oxygen of carbonyl group of the ligands are involved in coordination with metal ion. The IR spectra of the complexes show peaks in the range 3373–3529 and around 3203–3404 cm^−1^ assigned for the free O–H and N–H functional groups.

^1^H, ^13^C NMR spectra of L^1^ and L^2^ show the expected signals (see Section 2). ^1^H NMR of the ligands show peaks at chemical shift ca. 3.84 ppm. This singlet peak with three proton integration has been assigned to the methyl moiety of the methoxy group (3H, s, CH_3_). As expected this signal appeared downfield. The chemical shift for the O–H group was observed at 8.57 and 7.92 ppm for L^1^ and L^2^, respectively. The deshielding of this group and shifted downfield may be due to hydrogen bonding to the NMR solvent, which lead to decrease of the density of electrons on the hydroxyl group. Signals at 7.0–7.4 ppm were assigned to protons of aromatic ring. The chemical shift at 8.74 and 8.34 ppm in L^1^ and L^2^, respectively assigned to –CH=N–(imine) protons. 

### 4.3. Electronic Spectra and Magnetic Moment Measurements

The UV-Vis spectrum of L^1^ exhibits a high intense absorption peak at 376 nm, with a shoulder at 270 nm, assigned to n →π* and *π* → π*, respectively [28]. The spectrum for L^2^ exhibits a high intense absorption peak at 314 nm, with a shoulder at 261 nm, assigned to n →π* and *π* → π*, respectively. The electronic spectra of the cobalt(II) complex for 1 and 2 exhibit high intense peaks at 269 and 270 nm, respectively, due to ligand field. The absorption bands at 390 and 414 nm for 1 and 2, respectively, attributed to ^4^T_1_g^(F)^→ ^4^T_1_g^(P)^ transitions. The spectra of the Co(II) complex for 1 and 2 exhibited band which can be attributed to ^4^T_1_g^(F)^→ ^4^A_1_g^(F)^ transition, corresponding to tetrahedral Co(II) complexes [29–32]. The observed room temperature magnetic moment values 3.91 and 3.87 B.M for L^1^ and L^2^ Co-complexes, respectively confirmed their tetrahedral geometry. The slightly lower magnetic moment might be due to the slight deviation from the regular tetrahedral geometry. The spectra of the Cd(II) complex for 1 and 2 exhibited bands assigned to ligand *π* → π* and L → M charge transfer [29, 33]. The metal normally prefers tetrahedral coordination.

## 5. Antimicrobial Activity

The free Schiff-base ligands and their metal complexes were screened against *Bacillus *(G+) and* E. coli *(G−) to assess their potential as an antimicrobial agent by disc diffusion method. The measured zone of inhibition against the growth of various microorganisms is listed in Table 4. It is found that the ligand has higher antimicrobial activity than its metal complexes. This can be explained as follows. The biological activity of the Schiff-base ligand is related to the imine moiety, which plays a key role in the inhibition of the tested bacteria. The lower antimicrobial activity of the metal complexes compared with that in the ligand may be due to the strong interaction between the imine moieties and the metal ions. Such interaction will reduce the activity of the imine moiety in the inhibition.

## 6. Conclusion

In this paper, we have explored the synthesis and coordination chemistry of cobalt and cadmium complexes derived from the Schiff-base ligands L^1^ and L^2^. The complexes were prepared by mixing at reflux 2 mmole of the Schiff-base ligand with 1 mmole of the appropriate metal chloride. Complexes of the general formulae [M(L)_2_]Cl_2_ (where M = Co(II) and Cd(II); L = L^1^ and L^2^) were obtained. Physico-chemical analysis indicated the formation of four coordinate dicationic metal complexes. Biological activities revealed that the ligands have higher antimicrobial activity than their metal complexes.

## Figures and Tables

**Table 1:** Colours, yields, elemental analyses and molar conductance values.

Compound	Colour	Yield (%)	m.p	Found (Calc.) (%)					Λ_M_ (cm^2^ Ω^−1^ mol^−1^)
				M	C	H	N	Cl	
L^1^	Pale-yellow	54	183	—	58.97 (60.00)	4.47 (4.65)	10.39 (10.76)	—	—
[Co^II^ (L^1^)_2_]Cl_2_	Pale-green	55	216	8.12 (9.06)	47.49 (48.02)	3.48 (3.72)	8.21 (8.62)	9.55 (10.90)	70.2
[Cd^II^ (L^1^)_2_]Cl_2_	Yellow	46	288	14.20 (15.97)	43.78 (44.37)	3.19 (3.44)	7.77 (7.96)	9.12 (10.07)	71.4
L^2^	Orange-yellow	65	170	—	55.76 (56.51)	4.08 (4.38)	9.85 (10.14)	—	—
[Co^II^ (L^2^)_2_]Cl_2_	Light-orange	57	196	8.10 (8.64)	45.13 (45.76)	3.27 (3.54)	7.84 (8.21)	8.89 (10.39)	78.1
[Cd^II^ (L^2^)_2_]Cl_2_	Deep-yellow	43	270	14.04 (15.27)	42.09 (42.43)	3.12 (3.29)	7.28 (7.61)	8.54 (9.63)	75.3

**Table 2:** IR frequencies (cm^−1^) of the compounds.

Compound	*ν* (O–H)	*ν* (N–H)	*ν* (C = O)	*ν* (C=N)	*ν* (C–O)	*ν* (C–S)	*ν* (O–CH_3_)	*ν* (M–O)	*ν* (M–N)
L^1^	3473	3203	1664	1629	1271	—	1157	—	—
[Co^II^ (L^1^)_2_]Cl_2_	3529	3404	1641	1600	1257	—	1124	619	549
[Cd^II^ (L^1^)_2_]Cl_2_	3450	3226	1643	1613	1263	—	1126	698	540
L^2^	3375	3163	1669	1618	1265	846, 1361	1134	—	—
[Co^II^ (L^2^)_2_]Cl_2_	3448	3147	1647	1603	1203	817, 1303	1147	650	404
[Cd^II^ (L^2^)_2_]Cl_2_	3373	3209	1649	1608	1223	844, 1342	1149	678	416

**Table 3:** U.V-Vis spectral data in DMSO solutions.

Compound	*μ*_eff_ (BM)	Band position (*λ*nm)	Extinction coefficient *ε*_max⁡_ (dm^3^ mol^−1^ cm^−1^)	Assignments
L^1^		270	3773	*π* → π*
		376	147	*n* → π*

[Co^II^ (L^1^)_2_]Cl_2_		269	2797	*π* → π*
	3.91	490	75	^ 4^T_1_g^(F)^→^4^T_1_g^(P)^
		674	91	^ 4^T_1_g^(F)^→^4^A_2_g^(F)^

[Cd^II^ (L^1^)_2_]Cl_2_		294	587	*π* → π*
		330	682	CT

L^2^		261	3577	*π* → π*
		314	2466	*n* → π*

[Co^II^ (L^2^)_2_]Cl_2_		270	3737	*π* → π*
	3.87	424	154	^ 4^T_1_g^(F)^→^4^T_1_g^(P)^
		633	147	^ 4^T_1_g^(F)^→^4^A_2_g^(F)^

[Cd^II^ (L^2^)_2_]Cl_2_		269	888	*π* → π*
		302	1601	CT

**Table 4:** Antibacterial activities of the synthesised Schiff-bases and metal complexes.

Compounds	*Bacillus* (G+)		*E. coli* (G−)	
L^1^		++		+++
[Co^ II^ (L^1^)_2_]Cl_2_		+		+
[Cd^ II^ (L^1^)_2_]Cl_2_		−		+
L^2^		+		++
[Co^ II^ (L^2^)_2_]Cl_2_		+		−
[Cd^ II^ (L^2^)_2_]Cl_2_		−		−

^−^: No inhibition = inactive, ^+^: (2–4) mm = active, ^++^: (5–7) mm = more active, ^+++^: (8–13) mm = highly active.
